# Expanding the Distributed Generation Concept: Toward Decentralized Energy and Water Supply

**DOI:** 10.1002/gch2.201800006

**Published:** 2018-04-10

**Authors:** Rosaria Ciriminna, Mario Pecoraino, Francesco Meneguzzo, Mario Pagliaro

**Affiliations:** ^1^ Istituto per lo Studio dei Materiali Nanostrutturati CNR via U. La Malfa 153 90146 Palermo Italy; ^2^ via C. Giaquinto 14 90135 Palermo Italy; ^3^ Istituto di Biometeorologia CNR via G. Caproni 8 50145 Firenze Italy

**Keywords:** distributed generation, rainwater harvesting, solar energy, sustainable development goals, water scarcity

## Abstract

Rainwater harvesting decentralizes the water supply in full analogy to what building‐integrated photovoltaics and solar thermal do for electricity and heat. In this new decentralized generation scenario, the built environment is used to collect both sunlight photons and water molecules with significant economic and environmental benefits. Referring to recent progress and to real‐life findings from around the world, this work provides an answer to several relevant questions of direct interest to policy makers and early adopters of broadened distributed generation. The conclusions offer guidelines for developing a successful distributed generation strategy at the regional level by unifying solar energy and water management policies.

## 1 Introduction

Harvesting rainwater predates written published literature and has been practiced for millennia. Rainwater is the purest water in the natural environment (a few tens of ppm of total dissolved solids, depending on the topography, season, climate, and atmospheric pollution levels),^1^ and its catchment is a highly effective way to make water available for irrigation^2^ and for domestic water supply (drinking and washing).^3^ Identifying 170 articles published between 1970 and 1980 the authors of one of the first review articles on rainwater harvesting potential for enhanced crop production were reporting in 1982 that all revealed “an awareness of the increasing need for rainwater harvesting and a recognition of its potential.”^4^ Six years later, however, a public government scholar reviewing one of the first books^5^ describing progress and problems encountered in adopting rainwater harvesting in Africa, India, and Southeast Asia concluded the following:

In the case of domestic water supply via catched rainwater the diseconomies of scale in small tanks, the doubts of householders (and local authorities, in my experience) about the cleanliness of the water stored (an important area for extension education), and the fears of spreading mosquitoes and disease, have often put a brake on public and private investment in rainwater collection for domestic use.^6^

Regardless of its significant economic and environmental potential, the use of rainwater harvesting remained scattered and limited to specific situation until the late 1990s when China adopted it on large scale to make domestic and irrigation water available to farmers in the rural mountainous regions of the Gansu Province, solving a chronic water supply shortage for over 1.25 million people.^7^ Efforts were so successful that an annual *Rainwater Harvesting and Utilization* international training course was launched in 2003 by the Gansu Research Institute for Water Conservancy, followed in 2015 by an updated book including the resource materials developed for the said course.^8^

By early 2018, a search (excluding patents and citations) of “rainwater harvesting” in Google Scholar returned 31 200 results, with many studies reporting successful application of rainfall harvesting from around the world. Australia, for instance, has been using rainwater indoors for decades. South Korea's law requires since 2005 that all new buildings in the country's cities capture, store, and use rainwater. Canada, which along with Brazil and Russia is the world's leading country in terms of water reservoirs, had its housing agency publishing the first guidelines for residential rainwater harvesting systems in 2015.^9^ Even in Brazil, scholars lately published the first guidelines aimed at government to deploy policies to incentivize rainwater catchment systems;^10^ whereas in Texas, the State's water agency awards annually the three best rainwater harvesting projects in residential, commercial/industrial, and educational/governmental categories since 2007.^11^ In Mediterranean countries, recent efforts toward rainwater harvesting include its use as a control option to reduce runoff peaks,^12^ as well as the use of harvested rainwater in urban agriculture for food production.^13^

Even though in some countries (for example, the plumbing code in the United States) the law does not allow indoor use of harvested rainwater, scholars in the US recently found that rainwater harvesting could cover >50% of the domestic water demand in most country's counties,^14^ with low population density counties having the potential to meet their annual water needs capturing rainwater with existing rooftops, and high‐density counties having the potential to cover ≈20% of their annual demand.

The state of the art of rainwater harvesting systems including their design, construction, and management has been thoroughly reviewed in 2016,^15^ and excellent books have been lately published.^3^, ^8^ Referring to recent advances and findings from real‐life applications around the world, this work provides an answer to several relevant questions of direct interest to policy makers and tomorrow's adopters of broadened distributed generation. The conclusions offer guidelines for developing a successful distributed generation strategy at regional level by unifying solar energy and water policies, as early massive adoption of rainwater harvesting takes place concomitantly to the shift from centralized power generation toward distributed energy generation made possible by the photovoltaic (PV) global boom.^16^

## 2 Toward Decentralized Water and Energy Supply

Rainwater harvesting decentralizes the water supply in full analogy to what building‐integrated photovoltaics^17^ and building‐integrated solar thermal^18^ do for energy distributed generation, where electricity and heat are produced by collecting the sunlight photons hitting the solar collectors integrated on part of the building's external surface. The simple, yet powerful concept is to collect water and reuse it at an early stage in its natural cycle as rainfall runoff, reversing the conventional approach to “get rid of it as quickly as possible”^19^ which leads governments to building ever larger underground piping networks. The old approach is replaced by a new view in which rainwater is a valuable resource to be collected near where it has fallen via a simple collection system part of the built environment, using the resulting water near the collection site.^3^, ^8^ In general, the yearly amount of harvestable rainwater RH is given by Equation (1), 13

(1) $$\mathrm{RH}=P_{\mathrm{tot}}A_{\mathrm{catch}}C$$

where *A*_catch_ (in m^2^) is the surface area of the built environment (courtyards, rooftop, road, greenhouse, skylight, etc.) used to catch rainwater, *P*_tot_ is the total annual precipitation (in m), and *C* is the dimensionless runoff coefficient, namely, the efficiency of the harvesting system, whose value depends on both climatic and architectural factors varying between 0.6 (heavy rain and leaks, with gutters overflowing) and 0.95 (system is in perfect condition, no leaks and gentle rain).^20^ The overall amount of rainwater potentially intercepted and collected by rooftops in a certain urban area, for example, can be estimated by considering the overall rooftop size and the yearly average precipitation values from historic data.^13^

In rural areas, the approach to successful rainwater harvesting on large scale for irrigation in agriculture is that practically demonstrated by Chinese scholars in Gansu,^2^ namely, to make use of the freely available impermeable surfaces in the built environment, such as roofs, terrace, courtyards, roads, sport grounds, and greenhouses, to capture rainwater and divert the runoff with the aid of very simple gutters and downpipes guiding the collected water to an underground tank, installed with minimal skills and cost. A simple filter prevents sediment to enter the tank along with water. A pump (even and hand pump) is used to pump the harvested water through a distribution system.

The Chinese scholars were successful in meeting the most important requirement for widespread adoption of rainfall harvesting by rural households and farmers, namely, the low investment cost. This was achieved by developing a low‐cost approach in which the regional government provided for free the cement needed to self‐build two underground water tanks (30–50 m^3^ in volume) located next to the field crop, with each household irrigating one small piece of land (670 m^2^), feeding the drip irrigation lines with the stored rainwater via an hand pump.

By 2006, over 2.2 million subsurface water tanks had already been built, enabling irrigation of about 339 000 ha, with crop yield increases around 40%, on average, when compared to unirrigated crops.^21^

In general, rainwater collected from roofs is nonpotable due, for example, to bacterial content derived by the presence of feces from birds or other animals. It is relevant, therefore, to review how contemporary advanced rooftop harvesting systems were lately installed on the rooftop of 21 households in Accra, Ghana, to domestically produce potable water in a city where water purchased by tankers is the main source of drinking water for most citizens.^22^

**Figure 1:**
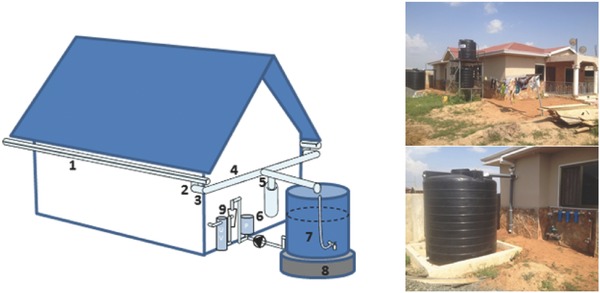
Domestic rainwater harvesting system providing potable water to 20 households in Ghana's Accra. 1 = gutters; 2 = downpipe; 3,4 = pipe bend; 5 = T‐piece; 6 = flush diverter; 7 = tank; 8 = cast foundation; 9 = distribution and disinfection system. Reproduced with permission.^22^ Copyright 2015, SINTEF.

In detail, in 2014 a public research organization from Norway installed the rainwater harvesting systems in collaboration with local companies. Some systems included a complete reverse osmosis purification unit incorporating ultraviolet disinfection and a filter. The roof, which does not include any lead‐containing roof paint, is used to collect water (**Figure** **1**). Gutters with a downward gradient of 2° carry on water toward a downpipe which incorporates a self‐cleaning grate to remove leaves and debris. A pipe bend transports water around the building's corners, and another from downpipe to tank, while a T‐piece connects to a first flush diverter whose task is to divert the initial volumes of dirty rainwater and ensure that the roof is clean before water is harvested for storage.

The harvested water is stored in a tank, preferably installed with cast foundation, from which it feeds an onward distribution and disinfection system with pressure‐pump, cartridge filter, and UV disinfection unit automatically maintaining a constant pressure in the supply pipes.

Remarkably, rather than focusing on the technical and economic aspects only, the team addressed the social dimension of rainwater harvesting since the early phase of the initiative, which included limited awareness and lack of supportive policies.^23^ In detail, in one year of monitoring (July 2014–July 2015) the rainwater harvesting systems, with one exception, met more than 90% of the household water needs, a percentage considerably higher than 72% estimated during the design phase thanks to relatively high measured rainfall (1217 mm) in place of an yearly average of 862 mm estimated on the basis of rainfall data over a 10‐year period.

Indeed, the performance of the systems even exceeded the best expectations in terms of convenience and water availability. Alas, due to frequent power outages, 38% of the samples did not comply with one or more of the criteria for bacteria counts of Ghana's water quality guidelines (**Table** **1**). However, water samples taken when UV disinfection was on, and the system was operated according to instruction, show that the system actually produced high‐quality potable water.

**Table 1:** Level of compliance with water quality standards (Adapted from ref. 23, with kind permission)

Sample	Physico‐chemical			Microbiological		
	No. of samples	No. of noncompliant samples	% of noncompliant samples	No. of samples	No. of noncompliant samples	% of noncompliant samples
ABL‐01	6	0	0	9	4	44
PAN‐01	0	–	–	2	2	100
BOR‐01	4	1	25	7	3	43
ADT‐01	4	0	0	8	1	13
KIS‐01	1	0	0	1	1	100
TEM‐01	3	0	0	9	4	44
OYI‐01	10	1	10	12	3	25

This single example shows the importance of having continuous access to electricity for having also access to high‐quality water, which is a condition perfectly met by using decentralized PV coupled to energy storage (for example in batteries), in all those regions and countries where either centralized power generation suffers from repeated outages or the power grid is simply absent.

**Figure 2:**
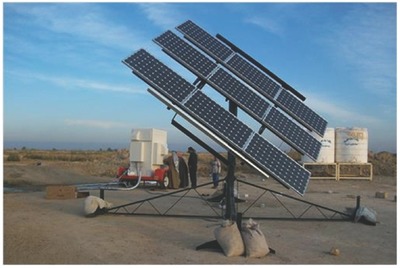
One of the solar‐powered water purification systems installed in Iraq between 2010 and 2012 to supply 729 rural villages and small cities with clean potable water after a cholera outbreak in 2009. Reproduced with permission.^24^ Copyright 2015, Solaropia.

Today's low‐cost PV modules make rainwater ultraviolet sterilization coupled to reverse osmosis or ultrafiltration convenient for widespread use in rural villages as well as in crowded cities. For example, in response to the dissemination of the cholera disease in Iraqi villages in 2009, the government asked an Iraqi firm, in partnership with a US‐based company, to install 729 solar PV‐powered water treatment units (**Figure** **2**) to supply the same number of villages and small cities with clean potable water from creeks and rivers (using ultrafiltration), or from brackish wells (using reverse osmosis).

The solar‐powered water purification units, with a capacity between 1 and 10 m^3^ h^−1^ (sufficient for villages and suburban areas with population from 500 to 10 000 people), were installed between 2010 and 2012, eventually deploying about 8 MW of solar PV power in what remains the world's largest solar water treatment project so far.^24^ The quality of the water obtained was comparable to bottled commercial water of high quality, being compliant with the stringent World Health Organization (WHO) water quality guidelines, i.e., significantly higher than that supplied to city residents across the world. The cholera epidemics quickly ended and the initiative was awarded the Energy Globe Award in 2015.^24^ Similarly, in the remote village of La Mancalona, in Mexico, plentiful clean water is now available for the 450 residents thanks to a solar‐powered reverse osmosis water purification system in which two PV modules (connected to batteries) power a set of pumps that push both brackish well water and collected rainwater through porous membranes that filter and purify the water prior to a final disinfection step with UV light.^25^

Potable water in this Yucatán forest village, prior to the installation of this broadened distributed generation system, was scarce. Now, along with health benefits, the system produces on average about 1000 L of clean water per day. The municipality sells for 5 pesos a 20 L bottle of high‐quality water to residents who previously had to pay 50 pesos for the same amount of water of much lower quality purchased from a distant facility (an hour of walking). The overall yearly revenue (about 49 000 pesos a year) is used both for the maintenance of the system and for the community.^26^

As the cost of PV, electrochemical energy storage and electricity‐powered water treatment technologies such as reverse osmosis and ultrafiltration continue to decrease, it is expected that low‐cost solar‐powered water purification systems using harvested rainwater, well, and brackish water will become ubiquitous across the world.

For comparison, a PV‐powered water purification unit able to process 1.5 L of water per minute, including stainless steel filter cartridges for removal of solid impurities from the rainwater and replaceable activated carbon cartridges for absorption of hazardous chemicals, was developed as early as of 1995.^27^ The system, which included a UV light disinfection unit for the inactivation of all waterborne pathogenic microorganisms (bacteria and viruses), was able to remove 99.9% bacteria on rainwater cistern water in Hawaii.^28^ At that time, however, the high cost of solar PV modules made the system barely affordable.

In general, recent real‐life studies applied to assess rainwater harvesting in terms of water quality, availability, and energy in residential buildings in megacities suffering from prolonged dry periods and groundwater depletion such as Dhaka (Bangladesh) confirm that the quality of water is satisfactory, with all main water quality parameters (pH, coliform bacteria, total dissolved solids, turbidity, ammonia, nitrates, lead, BOD_5_) meeting the Bangladesh standard for drinking water for several months of storage.^29^ For example, the entire water demand of a 60 persons' residential building (about 8100 L d^−1^) in 2010–2011 was met by a harvesting system with a catchment area of 172 m^2^ only.

**Figure 3:**
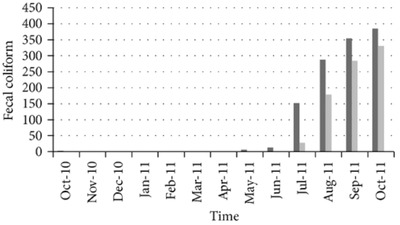
Variation of fecal coliform with time in the tank and flush harvested water in a 60 person residential building in Dhaka, Bangladesh Reproduced with permission.^29^ Published under the CC‐BY 3.0 license. Copyright 2014, The Authors.

However, the number of fecal coliforms present in the stored unused water suddenly and rapidly grew after a certain month (June 2011, **Figure** **3**) pointing to improper maintenance of tank and catchment areas, and thus to the need for proper maintenance to avoid the appearance of these harmful bacteria and ensure that water remains safe for household purposes.

Recent findings concerning the microbiological quality of roof‐harvested rainwater in American Samoa islands, where catchments are not yet fitted with first flush divert devices, revealed the presence of *Cryptosporidium* spp in 86% of the samples along with the frequent presence of opportunistic human pathogens such as *P. aeruginosa* and *M. intracellulare*, although at lower frequency than in tap water samples.^30^ The research team emphasized the need to educate practitioners of rainwater harvesting on the importance of regular cleaning and proper management of catchment areas and storage tanks to minimize health risks.

Even in remote areas with no access to electricity and to centralized distributed water systems, it is possible to access water of drinking quality by harvesting rainwater and then using low‐cost, yet highly effective water treatment techniques.

**Figure 4:**
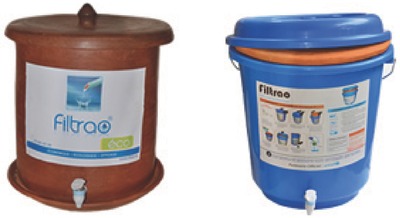
In 2016, the Filtrao Factory in Cote D'Ivoire produced 3000 ceramic water filters, many of which distributed in villages in Northern Cote D'Ivoire. Reproduced with permission.^32^ Copyright 2016, Filtrao Factory.

One is the ceramic filtration/disinfection system invented in 1981 by Mazariegos, and improved by Rivera in subsequent years. The filter is comprised of a porous ceramic incorporating in the finished ceramic filter biocidal colloidal silver particles. The ceramic is locally made out of clay mixed with sawdust or ground rice husks that on burning during clay firing leave tiny pores that block the passage of waterborne bacteria while filtering the water.^31^ Similar filters are produced in thousand exemplars every year at several ceramic filter companies around the world. One based in Cote D'Ivoire, for example, makes thousands of filters (**Figure** **4**) yearly since late 2010.^32^

Other disinfection techniques simply use direct solar radiation to kill microbiological contaminants. For example, in China's Gansu province farmers were given $20 parabolic solar heaters (cookers) to boil water and thus make it potable prior to domestic use, thereby avoiding burning of fossil or biomass fuels.

Similarly, in a rural community near Masaka, in southern Uganda, the quality of harvested rainwater at households was generally found to be unsafe for human consumption due to microbial contamination.^33^ It was enough to expose the harvested water stored in 2 L transparent plastic bottles to sunlight for 7 h (or for two days in case of cloudy days) to get water microbiologically safe for consumption. Even when air and water temperatures are low, indeed, the UV‐A rays in sunlight kill or inactivate germs such as viruses, bacteria, and parasites (*Giardia* and *Cryptosporidia*).^34^

The scholars who brought solar disinfection to that community registered enthusiastic feedback to training provided in aspects of sanitation, hygiene, and the use of solar disinfection. To encourage the use of solar disinfection, the same team suggested that plastic bottles need to be provided to households, though water quality concerns exist from long‐term leaching of toxic chemicals from the plastic bottles, which significantly increases with storage time and temperature.^35^

In general, high‐density urban areas will benefit more from rainwater harvesting than low‐density areas;^36^ with less water retrieved from the centralized water system, and lower amount of water entering the sewer network to eventually end up as wastewater requiring chemical and biological treatment in wastewater depurators.^37^

**Figure 5:**
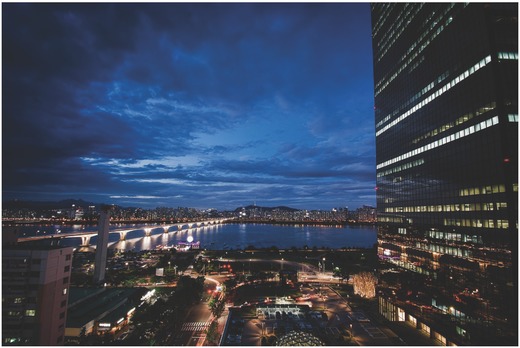
Even in a large city like Seoul, rainwater harvesting and management is carried out on township level in the Star City urban area (Reproduced from Pixabay, CC0 Creative Commons).

Large‐scale successful approaches include those demonstrated, for example, in South Korea or in Malaysia. In Seoul's (**Figure** **5**) Star City urban area, plentiful harvested rainwater is used for gardening, flushing the public toilet and to supply the firefighters with emergency water. Constructed between 2003 and 2006, the Star City 6.25 ha area includes numerous tall buildings up to 196 m high (35–58 floors above ground). Here, three large tanks with overall capacity of 3000 m^3^ (1000 m^3^ × 3 tanks) are refilled on the occasion of each precipitation tanks to rainwater from the roof (6200 m^2^) and terrace (45 000 m^2^) catchment areas.^38^

**Figure 6:**
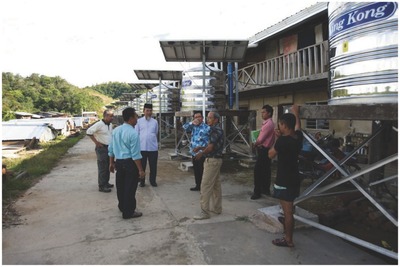
Malaysia's Long House rainwater harvesting project Reproduced with permission.^39^ Copyright 2012, H. A. Jamalluddin Shaaban.

Another instructive example is Malaysia's Long House rainwater harvesting project (**Figure** **6**) where since 2010 a total of 24 rainwater tanks (each of 1000 L) each supply a household with stored rainwater.^39^ Using simple diversion of the initial flush, the bacterial content is reduced and the stored water is used for toilets, bathing, mopping floors, washing clothes, and dish washing purposes, whereas the pumps are partly powered by solar PV modules.

The reliability of the rainwater capture system was about 87%, with observed monthly rainwater consumption of 285 m^3^ versus 248 m^3^ calculated based on assumption 66 L per person per day and average 5 persons per home (total daily water demand 8000 L).^39^

## 3 Energy Aspects

In terms of energy, recent findings concerning the embodied energy of harvested rainwater, namely, the ratio between the pumping requirements (product of the pump power per average usage time period) and the volume of rainwater pumped, is much lower than energy embodied in water delivered through the centralized water system which incorporates the electricity required for water catchment (from ground or surface sources), treatment, transport, and distribution. Scholars in Portugal for instance have developed an integrated model (*RaINvesT*) to assess the amount of embodied energy per unit of volume (kWh m^−3^) and the internal rate of return of investment for rainwater harvesting systems.^40^

The model was tested for a system installed at a university campus, revealing an embodied energy ratio of 0.013 kWh m^−3^, which is significantly lower than the embodied energy of water delivered through the centralized supply network (0.719 kWh m^−3^ in that case) due to the low pumping energy requirements.

In the same case study, the catchment system used the existent rainwater drainage system and an existing reservoir, affording a 9% internal rate of return. The cost of rainwater harvesting systems, in general, is mainly comprised of the tank/reservoir cost, followed, when present, by the rainwater purification unit.

## 4 Challenges and Recommendations

Looking forward to the near future in which the expanded distributed generation concept presented in this study will likely become ubiquitous, it is relevant to identify the remaining challenges to be addressed prior to widespread uptake of rainwater harvesting systems along with PV and solar thermal systems distributed in the built environment.

### 4.1 Renewed Education

The first need concerns expanded and renewed education of water and energy management professionals. As noted by Han, one of the world's leading scholars in the field, “rainwater harvesting *and* management is a new approach to solve many of the world's water problems.”^41^ Because of this relevance, education of professionals in rainwater harvesting needs to be expanded in distributed generation innovative courses whose focus is brought from centralized energy and water supply to distributed energy and water supply. Likewise to solar energy education whose programs and teaching methods need to be broadened and reshaped to serve several new societal and economic needs,^42^ new education in distributed generation integrates science, energy and water management, engineering, economics, and management topics.

The overall aim is to shape professionals capable to understand, develop, and disseminate the use of solar energy, energy efficiency, and rainwater harvesting seen as critical resources for the community through the numerous advantages they provide to the users of urban and extra‐urban buildings where most energy and water consumption actually takes place. In this new scenario in which surfaces of the built environment are used to collect both sunlight photons and water molecules, harvesting water in rainfall is leading to a renaissance of the urban water culture which, as noted by Krishnamurthy,^43^ mostly went lost with centralized water systems.

The newly formed professionals, in brief, are energy managers with an updated knowledge of rainwater harvesting, solar energy, and energy efficiency solutions who learned how to effectively promote said technologies also thanks to newly shaped skills and competences in management, communication, and sociology, now informing their daily practical activities.

Showing evidence of the integrated approach invoked herein and elsewhere^44^ for energy management education, the first *International Rainwater Harvesting Training Course for Developing Countries* held in 2003 in Lanzhou, capital of the Gansu Province, included in its topics economic evaluation and community management to effectively involve all farmers to actually take part into the program. Some 40 participants from 19 countries in Africa and Asia were trained for 45 d (20 d lecturing, 10 d field trips to rainwater harvesting projects, and 4 d evaluation) by some of the world's leading scholars in rainwater harvesting.^45^ As mentioned above, Chinese scholars (and China's central government which financed the course) were among the first to understand the national and international scope of the successful rainfall water harvesting program launched in the Gansu province in 1996. So significant were the improvements to water security, environmental management, and livelihoods that the Gansu experience was readily shared throughout the whole country. As a result, already in 2006 over 30 million people in 15 China's provinces, mainly in remote and mountainous areas, were benefiting from rainwater harvesting for both domestic water supply and crop production improvement.^2^

### 4.2 Community Involvement and Proactive Policies

Along with renewed education, large‐scale adoption of rainwater harvesting requires community involvement activities and government proactive action. In Malaysia, for instance, following a brief water crisis that occurred around 1998, the Government published the *Guidelines for Installing Rainwater Collection and Utilization System* already in 1999. Almost two decades later, reporting several successful case studies in the country,^39^ the director of Malaysia's hydraulic research institute emphasized how incorporation of rainwater harvesting systems in a housing area including commercial and public buildings made possible to achieve a 50% reduction of the peak discharge, calling for a comprehensive vision for which rainwater utilization should not be restricted to supplementing water supply to reduce the water bill, but also related to other aspects of living like food, water and energy security, flood mitigation, and environmental rehabilitation. Rainwater harvesting, indeed, reduces the (i) threat of local flooding, (ii) need for clean water in water distribution system, (iii) pollution of freshwater bodies via stormwater runoff, and (iv) energy and chemicals demand for wastewater treatment.^46^

Similarly, reporting the case of the wealthy Sant Cugat del Vallès Spain's city (56 000 inhabitants with very high individual water consumption rate) which pioneered the promotion of rainwater harvesting in residential buildings since 2002, scholars in Spain recently reported very high public acceptance and user satisfaction for water harvesting thanks to the high quality of the harvested rainwater due to integrated water treatment (filter, first flush diversion, and disinfection membranes).^47^

In general, government investment in distributed energy generation via today's low‐cost solar PV and solar thermal systems is more beneficial than investing in expanding centralized energy generation. Similarly, public investment in decentralized rainwater harvesting is more beneficial than investing in expanding centralized water supply systems. Incentives using taxpayer money is fully justified via the reduced costs of runoff water treatment as well as by the ever growing costs of the flooding consequences.

Incentives and favorable policies supporting rainwater capture systems will ideally get along with those supporting adoption of solar collectors for distributed generation. In economically developed countries, new legislation will ideally require all new buildings, and all buildings undergoing deep renovation, to be equipped with rainwater harvesting system as it happens in South Korea where, convinced by Seoul's Star City spectacular success, the Government passed new building regulations incorporating rainwater harvesting/stormwater management systems into new buildings.^48^

Similarly, governments will provide incentives to residential property owners installing or updating a rainwater harvesting system in existing or new homes by a rebate incentive program. This is, for example, what the Regional District of Nanaimo does in Canada, offering a total of $750 available per household for the purchase and installation of a rainwater harvesting system, comprised of a $450 rebate for a cistern rated for potable use and able to collect a minimum of 4546 L of water, and up to $300 available for other eligible expenses such as transport piping, debris traps, filters, and installation costs.^49^

Similar rebate programs exist in several other cities across economically developed regions of the world, with some rebates incentivizing rainwater harvesting systems to offset potable water demand, while others aim to reduce strain on stormwater drainage systems. In developing countries, governments will ideally support citizens co‐financing purchase of rainwater harvesting systems and treatment units in rural villages and small cities with dramatic health, social, and economic benefits. From Ghana to Cote D'Ivoire to Iraq, the examples cited in this study provide useful case studies.

In both developed and developing countries, farsighted governments will involve the above‐mentioned newly shaped distributed generation managers to launch educational programs for schoolchildren in schools who will be shown how solar collectors and rainwater harvesting systems used by the school actually reduce consumption of energy and water retrieved from the grid.

## 5 Outlook and Conclusions

From Spain to Ghana, from Malaysia to South Korea through several other countries, referring to real‐life findings from around the world, this work provides guidelines of direct interest to policy makers and early adopters of broadened distributed generation: the decentralized supply of energy and water. In general, all impermeable surfaces, including sidewalks, car parks, and urban and extra‐urban roads, can be used to collect rainwater easily guiding it to cisterns and reservoirs to be safely used for all nonpotable uses including irrigation.^3^, ^8^ Today's domestic rainwater harvesting systems, furthermore, can be easily equipped with reverse osmosis and ultrafiltration water depuration systems which, powered by electricity, convert harvested rainwater into high‐quality potable water generally much better than potable water retrieved from the public water networks.

This shows the importance to continuously access electricity also to continuously access potable water, as the example of homes equipped with such systems in Ghana's capitol presented above (Section 2) clearly demonstrates.^22^ Due to frequent power outages close to 40% of water samples supposed to be potable were found to be microbiologically contaminated, whereas water samples taken when UV disinfection was active (power was on) were comprised of high‐quality water even exceeding the WHO guidelines for water quality.

It is therefore remarkable, and consistent with the arguments of this study, that today's PV modules with their low cost (<$0.40 W) and high power output (easily exceeding 300 W and >400 W for bifacial modules that will become mainstream in a few years) provide the long sought‐after access to electricity to all those people currently lacking it, for example, in rural southern Asia, sub‐Saharan Africa, remote small islands (about 70% of Pacific islander households do not have access to electricity)^50^ and even certain areas of oil‐rich Middle East. For example, the 80 000 residents of a UN refugee camp in Zaatari, Jordan, since late 2017 access electricity for 14 h a day from previous 8 h, thanks to a 12.9 MW PV plant supplying electricity to homes, clinics, schools, and other facilities.^51^ The same is happening at fast pace in India where the capacity of 16.68 GW installed by the end of 2017 (when another 6.5 GW were under construction) was followed by auctions (bids) to install another 14 000 MW of solar projects only in the first three months of 2018.^52^ India, Bangladesh, Indonesia, the Philippines, and other large Asian countries with a large, young population are among those who will benefit more from adopting *en masse* the expanded distributed generation approach praised in this study.

Renewing and expanding the education of tomorrow's energy managers, involving the community, and assuming a proactive role at national and local level, the governments of both economically developed and developing countries will be able to accelerate the transition from centralized energy and water supply to the distributed generation scenario thereby freeing huge financial, energy, and environmental resources currently spent with centralized energy and water supply systems.

## Conflict of Interest

The authors declare no conflict of interest.
